# [μ-11,23-Dibromo-3,7,15,19-tetra­aza­tri­cyclo­[19.3.1.1^9,13^]hexa­cosa-1(25),2,7,9,11,13(26),14,19,21,23-deca­ene-25,26-diolato-κ^4^ *N* ^3^,*N* ^7^,*O*,*O*′:κ^4^ *O*,*O*′,*N* ^15^,*N* ^19^]bis[perchloratocopper(II)]

**DOI:** 10.1107/S1600536808000354

**Published:** 2008-01-11

**Authors:** Han-Ping Zhang, Jin-Xia Zhu, Hong Zhou, Zhi-Quan Pan

**Affiliations:** a Key Laboratory for Green Chemical Processes of the Ministry of Education, Wuhan Institute of Technology, Wuhan, 430073, People’s Republic of China; b Hubei Open Center for the Experimental Teaching of Fundamental Chemistry, Wuhan Institute of Technology, Wuhan, 430073, People’s Republic of China

## Abstract

The title complex, [Cu_2_(C_22_H_20_Br_2_N_4_O_2_)(ClO_4_)_2_], was prepared by the condensation of 2,6-diformyl-4-bromo­phenol with 1,3-diamino­propane in the presence of copper(II) ions. The macrocyclic ligand shows an approximately planar structure except for the two propene groups in the macrocycle. The coordination polyhedron of each Cu atom can be described as distorted square pyramidal. The two Cu atoms are bridged by two phenolate O atoms of the macrocycle, with a Cu⋯Cu distance of 3.109 (2) Å.

## Related literature

For related literature, see: Chen *et al.* (2005 ▶); Taniguchi (1984 ▶); Wang *et al.* (1997 ▶); Zhou *et al.* (2005 ▶); Mohanta *et al.* (1998 ▶); Wada *et al.* (1995 ▶).
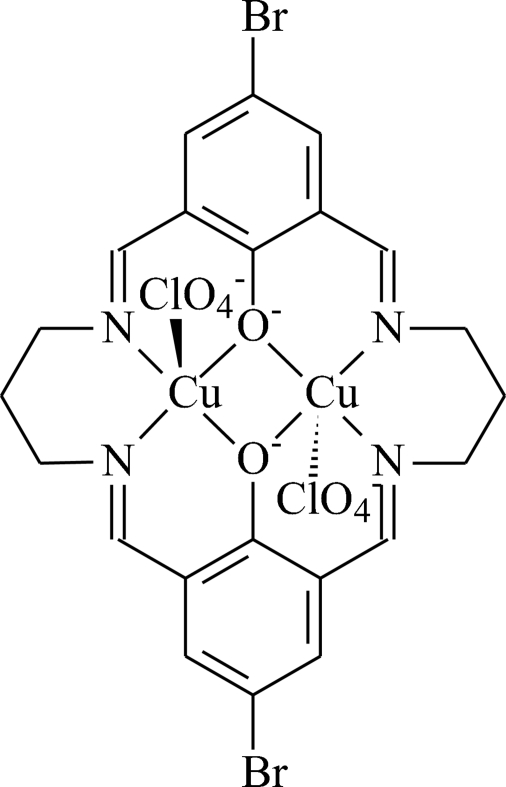


## Experimental

### 

#### Crystal data

[Cu_2_(C_22_H_20_Br_2_N_4_O_2_)(ClO_4_)_2_]
*M* *_r_* = 858.22
Monoclinic, 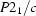

*a* = 15.7760 (18) Å
*b* = 8.6253 (10) Å
*c* = 21.501 (3) Å
β = 110.901 (2)°
*V* = 2733.2 (6) Å^3^
*Z* = 4
Mo *K*α radiation
μ = 4.74 mm^−1^
*T* = 191 (2) K
0.20 × 0.16 × 0.14 mm

#### Data collection

Bruker SMART APEX CCD diffractometer
Absorption correction: multi-scan (*SADABS*; Bruker, 2000 ▶) *T*_min_ = 0.42, *T*_max_ = 0.52
15132 measured reflections
5366 independent reflections
3505 reflections with *I* > 2σ(*I*)
*R*_int_ = 0.053

#### Refinement

*R*[*F* ^2^ > 2σ(*F* ^2^)] = 0.061
*wR*(*F* ^2^) = 0.138
*S* = 1.03
5366 reflections
379 parameters
H-atom parameters constrained
Δρ_max_ = 0.56 e Å^−3^
Δρ_min_ = −0.89 e Å^−3^

### 

Data collection: *SMART* (Bruker, 2000 ▶); cell refinement: *SAINT* (Bruker, 2000 ▶); data reduction: *SAINT*; program(s) used to solve structure: *SHELXTL* (Bruker, 2000 ▶); program(s) used to refine structure: *SHELXTL*; molecular graphics: *SHELXTL*; software used to prepare material for publication: *SHELXTL*.

## Supplementary Material

Crystal structure: contains datablocks global, I. DOI: 10.1107/S1600536808000354/hg2366sup1.cif

Structure factors: contains datablocks I. DOI: 10.1107/S1600536808000354/hg2366Isup2.hkl

Additional supplementary materials:  crystallographic information; 3D view; checkCIF report

## Figures and Tables

**Fig. 1.**
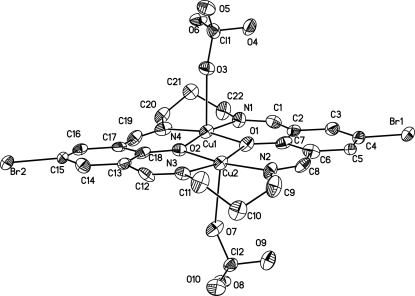
A view of the stucture of the title complex, showing the labeling of the non-H atoms and 30% probability ellipsoids; H atoms have been omitted for clarity.

## supplementary crystallographic information

### Comment

Schiff base macrocyclic ligands with two phenolic groups, capable of binding two metal ions in close coordination cavities simultaneously, are known to form various types of the transition metal complexes. These complexes can exhibit special optical, electric and magnetic properties (Mohanta *et al.*, 1998; Wang *et al.*, 1997).

Previous research shows that substituents on phenolic group has influences the structure and properties of these macrocyclic complexes. Much work have been done on this kind of complexes where the substituents in the phenolic group are found to be methyl, chlorine and *n*-butyl but few examples of bromide substituents are reported (Zhou *et al.*, 2005; Chen *et al.*, 2005).

In this work, a new dinuclear copper complex with Br substituent in phenolic group, [Cu_2_L(ClO_4_)_2_], was obtained and its crystal structure was determined by X-ray diffraction. Where, *L* denotes the above mentioned macrocyclic ligand. (Scheme).

A perspective view of the title complex is shown in Figure 1. The complex consist of two Cu(II) cations, one ligand and two perchlorate anions. The macrocyclic ligand exhibits an approximately planar structure. Each Cu atom has a slightly distorted square-pyramidal coordination with one O atom of a perchlorate anion in the apical position. Although the two Cu atom are in the same environment, there are small differences in the bond lengths and angles relevant to the copper coordination spheres. The base plane is composed of two imine N atoms and two phenolic O atoms with the mean plane deviation of 0.0227Å (for N1N4O1O2) and 0.0375Å (for N2N3O1O2), respectively. The lengths of Cu1—O3 and Cu2—O7 in the axial positions are 2.400 (6)Å and 2.421 (5) Å, respectively, that are somewhat larger than those of the bonds in the base plane, Cu—N or Cu—O (from 1.946 (8)Å to 1.982 (4) Å). Two Cu(II) ions in each center are located in the positions that slightly depart from relevant mean base planes towards the apical O atoms of perchlorate anions. (Distance from Cu1 to the center of the mean plane (I) of N1—N4—O1—O2 is 0.170 Å, Cu2 to the center of the mean plane(II) of N2—N3—O1—O2 is 0.169 Å.) The angles of axial Cu—O bonds with the relative mean planes (I) and (II) are 86.2° and 85.0°, respectively. Two perchlorate anions are located on opposite sides of the whole molecular plane. The presence of two bridging phenolic oxo atoms gives rise to a short metal-metal distance (Cu—Cu 3.109 (2) Å), typical for dinuclear complexes with macrocyclic phenoxo-bridging ligands.

### Experimental

2,6-Diformyl-4-bromophenol was prepared according to literature procedures (Taniguchi, 1984). The title complex was synthesized by the following procedure. A solution of 1,3-diaminopropane(0.111 g, 1.5 mmol) in absolute ethanol (15 ml) was added to an ethanol solution (15 ml) of 2,6-Diformyl-4-bromophenol (0.344 g, 1.5 mmol) under stirring. To the suspension of above mixture, copper perchlorate hexahydrate (0.556 g, 1.5 mmol) and 1 ml triethylamine were added and stired for 20 h at ambient temperature. The resulting green precipitate was filtered, washed with ethanol (2 × 30 ml) and dissolved in acetonitrile (15 ml). Single crystals suitable for X-ray diffraction were obtained by a slow diffusion of ethyl acetate into the acetonitrile solution.

### Refinement

All H atoms were placed in calculated positions, with C—H distances in the range of 0.93–0.97 Å, and included in the refinement in the riding-model approximation, with *U*_iso_(H) = 1.2–1.5 *U*_eq_(C/O).

### Crystal data

**Table:**

[Cu_2_(C_22_H_20_Br_2_N_4_O_2_)(ClO_4_)_2_]	*F*_000_ = 1688
*M**_r_* = 858.22	*D*_x_ = 2.086 Mg m^−^^3^
Monoclinic, *P*2_1_/*c*	Mo *K*α radiation λ = 0.71073 Å
Hall symbol: -P 2ybc	Cell parameters from 3876 reflections
*a* = 15.7760 (18) Å	θ = 2.6–26.0º
*b* = 8.6253 (10) Å	µ = 4.74 mm^−^^1^
*c* = 21.501 (3) Å	*T* = 191 (2) K
β = 110.901 (2)º	Block, dark green
*V* = 2733.2 (6) Å^3^	0.20 × 0.16 × 0.14 mm
*Z* = 4	

### Data collection

**Table:**

Bruker SMART APEX CCD diffractometer	5366 independent reflections
Radiation source: sealed tube	3505 reflections with *I* > 2σ(*I*)
Monochromator: graphite	*R*_int_ = 0.053
*T* = 191(2) K	θ_max_ = 26.0º
phi and ω scans	θ_min_ = 2.0º
Absorption correction: multi-scan(SADABS; Bruker, 2000)	*h* = −16→19
*T*_min_ = 0.42, *T*_max_ = 0.52	*k* = −10→10
15132 measured reflections	*l* = −25→26

### Refinement

**Table:**

Refinement on *F*^2^	Secondary atom site location: difference Fourier map
Least-squares matrix: full	Hydrogen site location: inferred from neighbouring sites
*R*[*F*^2^ > 2σ(*F*^2^)] = 0.061	H-atom parameters constrained
*wR*(*F*^2^) = 0.138	*w* = 1/[σ^2^(*F*_o_^2^) + (0.06*P*)^2^ + 1.55*P*] where *P* = (*F*_o_^2^ + 2*F*_c_^2^)/3
*S* = 1.03	(Δ/σ)_max_ < 0.001
5366 reflections	Δρ_max_ = 0.56 e Å^−^^3^
379 parameters	Δρ_min_ = −0.89 e Å^−^^3^
Primary atom site location: structure-invariant direct methods	Extinction correction: none

### Special details

**Table:**

Geometry. All e.s.d.'s (except the e.s.d. in the dihedral angle between two l.s. planes) are estimated using the full covariance matrix. The cell e.s.d.'s are taken into account individually in the estimation of e.s.d.'s in distances, angles and torsion angles; correlations between e.s.d.'s in cell parameters are only used when they are defined by crystal symmetry. An approximate (isotropic) treatment of cell e.s.d.'s is used for estimating e.s.d.'s involving l.s. planes.
Refinement. Refinement of *F*^2^ against ALL reflections. The weighted *R*-factor *wR* and goodness of fit *S* are based on *F*^2^, conventional *R*-factors *R* are based on *F*, with *F* set to zero for negative *F*^2^. The threshold expression of *F*^2^ > σ(*F*^2^) is used only for calculating *R*-factors(gt) *etc*. and is not relevant to the choice of reflections for refinement. *R*-factors based on *F*^2^ are statistically about twice as large as those based on *F*, and *R*- factors based on ALL data will be even larger.

### Fractional atomic coordinates and isotropic or equivalent isotropic displacement parameters (Å^2^)

**Table:**

	*x*	*y*	*z*	*U*_iso_*/*U*_eq_	
Br1	−0.09268 (5)	−0.42485 (9)	0.55710 (4)	0.0518 (2)	
Br2	0.61370 (5)	0.46431 (9)	0.43389 (4)	0.0483 (2)	
C1	0.2343 (5)	−0.2131 (8)	0.6347 (4)	0.0444 (18)	
H1	0.2347	−0.2747	0.6703	0.053*	
C2	0.1545 (5)	−0.2072 (7)	0.5796 (3)	0.0360 (15)	
C3	0.0825 (5)	−0.2996 (9)	0.5909 (4)	0.0498 (19)	
H3	0.0940	−0.3503	0.6313	0.060*	
C4	−0.0015 (5)	−0.3105 (9)	0.5415 (4)	0.0436 (17)	
C5	−0.0193 (5)	−0.2402 (8)	0.4820 (4)	0.0442 (17)	
H5	−0.0761	−0.2506	0.4487	0.053*	
C6	0.0491 (5)	−0.1503 (9)	0.4703 (3)	0.0446 (18)	
C7	0.1345 (5)	−0.1374 (8)	0.5174 (3)	0.0429 (17)	
C8	0.0233 (5)	−0.0779 (7)	0.4047 (3)	0.0386 (16)	
H8	−0.0362	−0.0972	0.3769	0.046*	
C9	0.0129 (5)	0.0570 (10)	0.3100 (4)	0.056 (2)	
H9A	−0.0293	−0.0252	0.2882	0.067*	
H9B	−0.0229	0.1463	0.3129	0.067*	
C10	0.0641 (5)	0.0982 (10)	0.2672 (3)	0.0509 (19)	
H10A	0.0226	0.1258	0.2231	0.061*	
H10B	0.1002	0.0104	0.2630	0.061*	
C11	0.1244 (5)	0.2319 (9)	0.2975 (4)	0.055 (2)	
H11A	0.0889	0.3087	0.3105	0.066*	
H11B	0.1433	0.2784	0.2635	0.066*	
C12	0.2833 (4)	0.2512 (10)	0.3632 (3)	0.0429 (17)	
H12	0.2833	0.2984	0.3243	0.052*	
C13	0.3678 (4)	0.2573 (8)	0.4152 (3)	0.0360 (15)	
C14	0.4363 (5)	0.3465 (8)	0.4044 (3)	0.0423 (17)	
H14	0.4238	0.4036	0.3654	0.051*	
C15	0.5216 (5)	0.3476 (8)	0.4523 (3)	0.0405 (17)	
C16	0.5422 (5)	0.2665 (8)	0.5085 (3)	0.0372 (15)	
H16	0.6011	0.2683	0.5394	0.045*	
C17	0.4748 (5)	0.1777 (8)	0.5214 (3)	0.0390 (16)	
C18	0.3842 (5)	0.1748 (8)	0.4747 (3)	0.0374 (16)	
C19	0.5020 (6)	0.0874 (9)	0.5857 (4)	0.052 (2)	
H19	0.5640	0.0747	0.6079	0.063*	
C20	0.5105 (5)	−0.0086 (9)	0.6818 (4)	0.053 (2)	
H20A	0.5556	0.0724	0.6988	0.064*	
H20B	0.5430	−0.1039	0.6814	0.064*	
C21	0.4607 (5)	−0.0294 (9)	0.7317 (4)	0.054 (2)	
H21A	0.5031	−0.0600	0.7750	0.064*	
H21B	0.4316	0.0666	0.7365	0.064*	
C22	0.3907 (6)	−0.1550 (11)	0.7029 (4)	0.063 (2)	
H22A	0.4236	−0.2450	0.6965	0.076*	
H22B	0.3673	−0.1822	0.7375	0.076*	
Cl1	0.25784 (12)	0.2959 (2)	0.66515 (9)	0.0465 (4)	
Cl2	0.22902 (11)	−0.2790 (2)	0.33413 (9)	0.0433 (4)	
Cu1	0.32546 (6)	−0.00913 (10)	0.56944 (4)	0.0405 (2)	
Cu2	0.19480 (5)	0.05601 (9)	0.42474 (4)	0.0350 (2)	
N1	0.3106 (4)	−0.1370 (8)	0.6406 (3)	0.0492 (15)	
N2	0.0670 (4)	0.0052 (7)	0.3793 (3)	0.0433 (15)	
N3	0.2095 (4)	0.1969 (6)	0.3582 (3)	0.0355 (12)	
N4	0.4549 (5)	0.0307 (8)	0.6115 (3)	0.0521 (17)	
O1	0.2005 (3)	−0.0499 (5)	0.5072 (2)	0.0401 (11)	
O2	0.3201 (3)	0.0963 (5)	0.4862 (2)	0.0375 (10)	
O3	0.2773 (4)	0.2128 (6)	0.6155 (3)	0.0512 (13)	
O4	0.1906 (4)	0.2219 (6)	0.6771 (3)	0.0582 (15)	
O5	0.2216 (4)	0.4368 (7)	0.6433 (3)	0.0577 (14)	
O6	0.3286 (3)	0.3008 (6)	0.7254 (3)	0.0534 (14)	
O7	0.2527 (3)	−0.1555 (6)	0.3774 (3)	0.0513 (14)	
O8	0.2945 (3)	−0.3882 (6)	0.3531 (3)	0.0532 (14)	
O9	0.1471 (4)	−0.3245 (6)	0.3311 (3)	0.0531 (14)	
O10	0.2198 (4)	−0.2300 (6)	0.2715 (3)	0.0551 (14)	

### Atomic displacement parameters (Å^2^)

**Table:**

	*U*^11^	*U*^22^	*U*^33^	*U*^12^	*U*^13^	*U*^23^
Br1	0.0498 (5)	0.0487 (4)	0.0545 (4)	−0.0148 (4)	0.0156 (4)	−0.0125 (4)
Br2	0.0449 (4)	0.0445 (4)	0.0554 (5)	−0.0174 (3)	0.0177 (4)	−0.0166 (3)
C1	0.047 (4)	0.043 (4)	0.042 (4)	0.020 (3)	0.015 (4)	0.010 (3)
C2	0.050 (4)	0.020 (3)	0.040 (4)	0.002 (3)	0.018 (3)	0.003 (3)
C3	0.047 (5)	0.051 (5)	0.049 (4)	−0.002 (4)	0.014 (4)	−0.010 (4)
C4	0.030 (4)	0.050 (4)	0.046 (4)	−0.003 (3)	0.009 (3)	−0.013 (3)
C5	0.034 (4)	0.047 (4)	0.054 (4)	−0.007 (3)	0.020 (3)	−0.024 (4)
C6	0.043 (4)	0.048 (4)	0.037 (4)	0.020 (3)	0.008 (3)	0.008 (3)
C7	0.048 (4)	0.036 (4)	0.034 (4)	0.008 (3)	0.000 (3)	−0.005 (3)
C8	0.048 (4)	0.027 (3)	0.027 (3)	−0.005 (3)	−0.003 (3)	0.002 (3)
C9	0.030 (4)	0.057 (5)	0.062 (5)	−0.001 (3)	−0.005 (4)	0.013 (4)
C10	0.050 (4)	0.060 (5)	0.029 (4)	0.000 (4)	−0.003 (3)	0.013 (3)
C11	0.042 (4)	0.056 (5)	0.048 (5)	0.004 (4)	−0.007 (4)	0.017 (4)
C12	0.025 (3)	0.079 (5)	0.025 (3)	0.008 (4)	0.009 (3)	−0.006 (3)
C13	0.034 (4)	0.033 (3)	0.040 (4)	−0.002 (3)	0.012 (3)	−0.011 (3)
C14	0.046 (4)	0.045 (4)	0.038 (4)	0.006 (3)	0.018 (3)	0.008 (3)
C15	0.043 (4)	0.042 (4)	0.042 (4)	−0.021 (3)	0.023 (3)	−0.019 (3)
C16	0.031 (4)	0.040 (4)	0.029 (3)	0.002 (3)	−0.002 (3)	−0.012 (3)
C17	0.043 (4)	0.036 (3)	0.038 (4)	0.004 (3)	0.014 (3)	−0.013 (3)
C18	0.038 (4)	0.029 (3)	0.035 (4)	0.007 (3)	0.000 (3)	−0.006 (3)
C19	0.043 (5)	0.046 (5)	0.051 (5)	−0.003 (4)	−0.005 (4)	−0.002 (4)
C20	0.042 (4)	0.048 (4)	0.045 (4)	0.000 (3)	−0.014 (4)	0.005 (3)
C21	0.046 (5)	0.057 (5)	0.043 (4)	0.014 (4)	−0.002 (4)	0.015 (4)
C22	0.052 (5)	0.074 (6)	0.044 (5)	−0.009 (4)	−0.006 (4)	0.014 (4)
Cl1	0.0314 (9)	0.0590 (12)	0.0457 (10)	−0.0003 (8)	0.0095 (8)	−0.0014 (8)
Cl2	0.0343 (9)	0.0461 (10)	0.0434 (10)	−0.0026 (7)	0.0066 (8)	−0.0110 (8)
Cu1	0.0354 (5)	0.0367 (5)	0.0385 (5)	0.0055 (4)	−0.0003 (4)	0.0015 (3)
Cu2	0.0271 (4)	0.0294 (4)	0.0384 (4)	0.0070 (3)	−0.0006 (3)	0.0026 (3)
N1	0.047 (4)	0.054 (4)	0.039 (3)	−0.006 (3)	0.006 (3)	0.005 (3)
N2	0.026 (3)	0.045 (3)	0.045 (3)	−0.003 (2)	−0.005 (3)	−0.002 (3)
N3	0.039 (3)	0.039 (3)	0.027 (3)	0.007 (3)	0.010 (3)	0.002 (2)
N4	0.046 (4)	0.053 (4)	0.040 (4)	0.012 (3)	−0.006 (3)	−0.007 (3)
O1	0.040 (3)	0.040 (3)	0.028 (2)	0.000 (2)	−0.003 (2)	0.005 (2)
O2	0.039 (3)	0.040 (3)	0.030 (2)	0.004 (2)	0.009 (2)	0.000 (2)
O3	0.051 (3)	0.050 (3)	0.053 (3)	0.009 (2)	0.020 (3)	−0.004 (2)
O4	0.055 (3)	0.057 (3)	0.059 (3)	−0.020 (3)	0.015 (3)	−0.009 (3)
O5	0.054 (3)	0.060 (3)	0.058 (3)	0.020 (3)	0.018 (3)	0.005 (3)
O6	0.042 (3)	0.054 (3)	0.050 (3)	0.016 (2)	0.000 (2)	−0.013 (2)
O7	0.031 (3)	0.038 (3)	0.063 (3)	0.013 (2)	−0.011 (2)	−0.008 (2)
O8	0.041 (3)	0.060 (3)	0.052 (3)	0.016 (3)	0.009 (3)	−0.017 (3)
O9	0.052 (3)	0.041 (3)	0.055 (3)	−0.006 (2)	0.005 (3)	0.015 (2)
O10	0.058 (4)	0.051 (3)	0.055 (3)	0.017 (3)	0.018 (3)	0.005 (3)

### Geometric parameters (Å, °)

**Table:**

Br1—C4	1.871 (8)	C16—C17	1.416 (10)
Br2—C15	1.922 (6)	C16—H16	0.9300
C1—N1	1.337 (10)	C17—C18	1.424 (9)
C1—C2	1.387 (10)	C17—C19	1.509 (10)
C1—H1	0.9300	C18—O2	1.312 (8)
C2—C7	1.396 (9)	C19—N4	1.180 (10)
C2—C3	1.477 (10)	C19—H19	0.9300
C3—C4	1.374 (10)	C20—N4	1.490 (9)
C3—H3	0.9300	C20—C21	1.550 (11)
C4—C5	1.352 (11)	C20—H20A	0.9700
C5—C6	1.421 (10)	C20—H20B	0.9700
C5—H5	0.9300	C21—C22	1.513 (12)
C6—C7	1.370 (10)	C21—H21A	0.9700
C6—C8	1.463 (9)	C21—H21B	0.9700
C7—O1	1.366 (9)	C22—N1	1.487 (9)
C8—N2	1.247 (9)	C22—H22A	0.9700
C8—H8	0.9300	C22—H22B	0.9700
C9—C10	1.468 (11)	Cl1—O4	1.339 (5)
C9—N2	1.498 (9)	Cl1—O5	1.354 (6)
C9—H9A	0.9700	Cl1—O6	1.376 (5)
C9—H9B	0.9700	Cl1—O3	1.408 (5)
C10—C11	1.489 (11)	Cl2—O9	1.330 (6)
C10—H10A	0.9700	Cl2—O8	1.349 (5)
C10—H10B	0.9700	Cl2—O10	1.368 (6)
C11—N3	1.532 (8)	Cl2—O7	1.375 (5)
C11—H11A	0.9700	Cu1—N4	1.947 (7)
C11—H11B	0.9700	Cu1—N1	1.966 (6)
C12—N3	1.222 (8)	Cu1—O1	1.980 (5)
C12—C13	1.403 (9)	Cu1—O2	1.983 (4)
C12—H12	0.9300	Cu1—O3	2.400 (5)
C13—C18	1.405 (9)	Cu2—N2	1.951 (6)
C13—C14	1.411 (9)	Cu2—N3	1.953 (5)
C14—C15	1.373 (10)	Cu2—O1	1.968 (5)
C14—H14	0.9300	Cu2—O2	1.977 (5)
C15—C16	1.332 (10)	Cu2—O7	2.421 (5)
			
N1—C1—C2	125.0 (7)	C21—C20—H20A	107.8
N1—C1—H1	117.5	N4—C20—H20B	107.8
C2—C1—H1	117.5	C21—C20—H20B	107.8
C1—C2—C7	131.3 (7)	H20A—C20—H20B	107.2
C1—C2—C3	110.9 (6)	C22—C21—C20	106.0 (7)
C7—C2—C3	117.7 (6)	C22—C21—H21A	110.5
C4—C3—C2	119.7 (7)	C20—C21—H21A	110.5
C4—C3—H3	120.1	C22—C21—H21B	110.5
C2—C3—H3	120.1	C20—C21—H21B	110.5
C5—C4—C3	121.3 (7)	H21A—C21—H21B	108.7
C5—C4—Br1	119.5 (5)	N1—C22—C21	123.8 (7)
C3—C4—Br1	119.2 (6)	N1—C22—H22A	106.4
C4—C5—C6	119.7 (7)	C21—C22—H22A	106.4
C4—C5—H5	120.1	N1—C22—H22B	106.4
C6—C5—H5	120.1	C21—C22—H22B	106.4
C7—C6—C5	121.5 (7)	H22A—C22—H22B	106.5
C7—C6—C8	122.7 (7)	O4—Cl1—O5	103.3 (4)
C5—C6—C8	115.8 (7)	O4—Cl1—O6	105.5 (4)
O1—C7—C6	122.0 (6)	O5—Cl1—O6	113.6 (4)
O1—C7—C2	117.9 (6)	O4—Cl1—O3	107.7 (3)
C6—C7—C2	120.0 (7)	O5—Cl1—O3	111.9 (3)
N2—C8—C6	131.3 (7)	O6—Cl1—O3	113.9 (3)
N2—C8—H8	114.3	O9—Cl2—O8	115.7 (4)
C6—C8—H8	114.3	O9—Cl2—O10	106.4 (3)
C10—C9—N2	116.7 (6)	O8—Cl2—O10	108.3 (3)
C10—C9—H9A	108.1	O9—Cl2—O7	106.7 (4)
N2—C9—H9A	108.1	O8—Cl2—O7	110.0 (3)
C10—C9—H9B	108.1	O10—Cl2—O7	109.5 (4)
N2—C9—H9B	108.1	N4—Cu1—N1	97.7 (3)
H9A—C9—H9B	107.3	N4—Cu1—O1	166.5 (3)
C9—C10—C11	108.8 (7)	N1—Cu1—O1	93.4 (2)
C9—C10—H10A	109.9	N4—Cu1—O2	92.2 (3)
C11—C10—H10A	109.9	N1—Cu1—O2	168.8 (2)
C9—C10—H10B	109.9	O1—Cu1—O2	76.07 (19)
C11—C10—H10B	109.9	N4—Cu1—O3	95.8 (2)
H10A—C10—H10B	108.3	N1—Cu1—O3	89.0 (2)
C10—C11—N3	116.7 (6)	O1—Cu1—O3	92.03 (19)
C10—C11—H11A	108.1	O2—Cu1—O3	95.11 (18)
N3—C11—H11A	108.1	N2—Cu2—N3	98.3 (3)
C10—C11—H11B	108.1	N2—Cu2—O1	93.1 (2)
N3—C11—H11B	108.1	N3—Cu2—O1	165.9 (2)
H11A—C11—H11B	107.3	N2—Cu2—O2	169.2 (2)
N3—C12—C13	133.6 (7)	N3—Cu2—O2	91.6 (2)
N3—C12—H12	113.2	O1—Cu2—O2	76.50 (19)
C13—C12—H12	113.2	N2—Cu2—O7	95.6 (2)
C12—C13—C18	121.3 (6)	N3—Cu2—O7	90.0 (2)
C12—C13—C14	117.1 (6)	O1—Cu2—O7	97.26 (19)
C18—C13—C14	121.6 (6)	O2—Cu2—O7	88.63 (17)
C15—C14—C13	118.9 (6)	C1—N1—C22	118.8 (6)
C15—C14—H14	120.6	C1—N1—Cu1	123.7 (5)
C13—C14—H14	120.6	C22—N1—Cu1	117.4 (5)
C16—C15—C14	122.3 (6)	C8—N2—C9	113.5 (6)
C16—C15—Br2	120.3 (5)	C8—N2—Cu2	123.0 (5)
C14—C15—Br2	117.4 (5)	C9—N2—Cu2	123.5 (5)
C15—C16—C17	120.3 (6)	C12—N3—C11	121.2 (6)
C15—C16—H16	119.8	C12—N3—Cu2	122.1 (5)
C17—C16—H16	119.8	C11—N3—Cu2	116.7 (5)
C16—C17—C18	120.4 (6)	C19—N4—C20	109.2 (7)
C16—C17—C19	118.1 (6)	C19—N4—Cu1	125.9 (6)
C18—C17—C19	121.5 (7)	C20—N4—Cu1	124.9 (6)
O2—C18—C13	122.0 (6)	C7—O1—Cu2	127.6 (4)
O2—C18—C17	121.6 (6)	C7—O1—Cu1	128.4 (4)
C13—C18—C17	116.5 (7)	Cu2—O1—Cu1	103.9 (2)
N4—C19—C17	128.6 (7)	C18—O2—Cu2	128.3 (4)
N4—C19—H19	115.7	C18—O2—Cu1	128.2 (4)
C17—C19—H19	115.7	Cu2—O2—Cu1	103.5 (2)
N4—C20—C21	117.9 (6)	Cl1—O3—Cu1	155.0 (3)
N4—C20—H20A	107.8	Cl2—O7—Cu2	144.6 (3)
